# Tension Monitoring of Wedge Connection Using Piezoceramic Transducers and Wavelet Packet Analysis Method

**DOI:** 10.3390/s20020364

**Published:** 2020-01-08

**Authors:** Xiaoyu Zhang, Liuyu Zhang, Laijun Liu, Linsheng Huo

**Affiliations:** 1 Engineering Research Center for Large Highway Structure Safety of the Ministry of Education, Chang’an University, Xi’an 710064, China; xiaoyuzhang@chd.edu.cn; 2 State Key Laboratory of Coastal and Offshore Engineering, Dalian University of Technology, Dalian 116024, China

**Keywords:** piezoceramics, prestress monitoring, steel strand, wedge anchorage connection, wavelet packet analysis

## Abstract

A steel strand is widely used in long span prestressed concrete bridges. The safety and stability of a steel strand are important issues during its operation period. A steel strand is usually subjected to various types of prestress loss which loosens the anchorage system, negatively impacting the stability of the structure and even leading to severe accidents. In this paper, the authors propose a wavelet packet analysis method to monitor the looseness of the wedge anchorage system by using stress wave-based active sensing. As a commonly used piezoceramic material, lead zirconate titanate (PZT) is employed with a strong piezoelectric effect. In the proposed active sensing approach, PZT patches are used as sensors and actuators to monitor the steel strand looseness. The anchorage system consists of the steel strand, wedges and barrel, which forms two different direct contact surfaces to monitor the tension force. PZT patches are pasted on the surface of each steel strand, corresponding wedge and barrel, respectively. Different combinations of PZTs are formed to monitor the anchoring state of the steel strand according to the position of the PZT patches. In this monitoring method of two contact surfaces, one PZT patch is used as an actuator to generate a stress wave and the other corresponding PZT patch is used as a sensor to detect the propagated waves through the wedge anchorage system. The function of these two PZTs were exchanged with the changing of transmission direction. The wavelet packet analysis method is utilized to analyze the transmitted signal between PZT patches through the steel strand anchorage system. Compared with the wavelet packet energy of received signals under different PZT combinations, it could be found that the wavelet packet energy increased with the increasing of anchorage system tightness. Therefore, the wavelet packet energy of received signal could be used to monitor the tightness of the steel strand during operation. Additionally, the wavelet packet energy of the received signals are different when the same PZT combination exchanges the energy transfer direction. With the comparison on the received signals of different combinations of PZTs, the optimal energy transfer path corresponding to different contact surfaces of the steel strand could be determined and the optimal experimental results are achieved.

## 1. Introduction

A steel strand is widely used in prestressed structures due to beneficial features such as a large cross-section area, softness and convenient location, high strength and low relaxation. As a skeleton component in the prestressed structure, the tension of the prestressed steel strand directly affects the durability and overall safety of these structures. However, due to the tensioning process, material properties and environmental conditions, prestress loss will occur in the steel strands, which will reduce the bearing capacity of the structure and bring potential or even serious harm to the overall safety of the structure [1,2].

In recent years, a series of accidents of prestressed bridge failures have occurred, most of which are caused by the loss of the prestress. During the operation of large-span bridges, problems such as excessive deflection of the main span and the cracks in the box girder due to the prestress tension not reaching the design value can be caused easily [3,4,5]. If the steel strand fails in the prestressed member, the member may be destroyed quickly without any signs on the surface. For a continuous multi-span unbonded prestressed structure, once one span of the prestressed steel strand fails, the remaining span of prestressed steel strands will fail together, resulting in overall structural damage and huge losses. Therefore, prestress monitoring of the steel strand is very important, and its non-destructive and in-service measurement has received extensive attention worldwide [6,7]. At present, there are mainly the vibration frequency method, resistance strain gauge method, pressure transducer method, magnetic flux leakage method, optical fiber detection method and magnetoelastic method for steel strand detection, but they have certain limitations [8,9,10]. Many methods can only detect the accessible components, such as external prestressed steel strands in concrete or bridge cables, and their application scope is greatly limited. Some detection methods are too expensive and few measurement points are arranged in the actual bridge-monitoring process. Meanwhile, most of the measurement sensors have a limited life span and cannot be replaced later or the replacement process is complex. In recent years, with the development of research on the propagation characteristics of an ultrasonic guided wave in a steel strand, the correlation between the propagation characteristics of the guided wave in the steel strand and the axial tension has been gradually explored [11,12,13,14,15]. Based on the simplified wire-to-wire contact model, Treyssède [16] used a semi-analytic finite element method to obtain the dispersion curve of the guided wave in the steel strand. Raišutis [17] conducted ultrasonic guided wave propagation experiments in multiple-wire ropes with artificial defects, successfully identified the damaged strands, and verified the feasibility of ultrasonic guided waves in the defect identification of steel strand. Moustafa [18] introduced fractal theory to evaluate the corrosion of steel strands through the fractal characteristics of guided wave signals under different degrees of corrosion, and proposed an outlier algorithm to improve the accuracy of the corrosion detection method. Xu [19] used a magnetostrictive ultrasonic guided wave to detect multiple broken wires in the same steel wire of a steel strand. Li [20] determined the influence of propagation length and concrete state on acoustic emission (AE) strength by comparing the attenuation of AE strength of the steel strand in concrete under four different environments. Based on ant colony optimization and self-organizing feature mapping technology, Li [21] identified the stress corrosion pattern of the steel strand and determined AE feature parameters by analyzing AE characteristic signals at different stress corrosion stages of the steel strand. When the guided wave propagates in the structure, the phase velocity will change with the frequency, which is called the dispersion phenomenon. The dispersion of the guided wave makes the signal width wider and the amplitude lower after the guided wave propagates to a certain distance. It is difficult to analyze the signal. The mode with larger dispersion is not suitable for waveguide detection. Therefore, the dispersion characteristics of an ultrasonic guided wave greatly affect the detection effect [22,23]. Alireza [24] built an ultrasonic guided wave detection system for corrosion damage of steel strands. Two broadband piezoelectric transducers were used to excite and receive ultrasonic guided waves. The transmission wave detection method was used to study the guided wave response during the corrosion of steel strands. The cross-sectional area loss is estimated by using a parameterless graph formed by the dispersion curve and wave velocity measurement. However, the current research results show that the guided wave propagation in the steel strand carries obvious tensile force information, but it is still difficult to find the propagation characteristics that can represent the tensile force and have strong engineering applicability from the complex guided wave signals. Therefore, a new non-destructive testing method is urgently needed for the health assessment of steel strands under different working conditions.

A piezoelectric lead zirconate titanate (PZT)-based technique is often used in active monitoring the safety state of structures over their entire life cycle due to great features such as their broadband frequency response and the ability of being employed as actuators or sensors [25,26,27]. The recent research has shown that low cost PZT patches can be used for vibration detection [28,29], energy harvesting [30], stress wave generation [31,32], damage detection [33,34] and structural health monitoring [35,36,37,38].

Wavelet packet analysis, a good signal-processing tool, is utilized by many researchers to monitor safety states or detect damage in structures. Xu applied PZT-based intelligent aggregate (SAs) as actuators, using piezoelectrics on the surface of the steel tube as sensors [39]. The artificially manufactured debonding areas of the concrete inside the steel tube can be monitored according to the evaluation index. Feng embedded smart aggregates based on PZT properties into reinforced concrete pipelines [40]. The cracking of a concrete pipeline can be monitored according to the energy attenuation at the crack location of the concrete pipeline. Zhang bonded PZT patches on the cross bars and vertical bar of cuplok scaffold, respectively. The propagated energy value increase with the tightness of cuplock connection, and the safety status of cuplock scaffold can be monitored [41]. Li attached PZT patches to surface of the pin and steel plate base, and the energy of different tension states is consistent with the results of a 3D finite element calculation. The experimental result proved that wavelet packet analysis method can be used to monitor the safety of the pin connection structures [42].

In this paper, an active monitoring method for prestress with a wedge connection based on the wavelet packet analysis method using piezoceramics was proposed. According to the composition characteristics of wedge anchorage system, two direct contact surfaces were formed. In the proposed active monitoring method, three PZT patches were attached to the surface of the steel strand, wedge and barrel, respectively. Once a certain contact surface was selected for prestress monitoring, two PZT patches corresponding to the selected contact surface formed a monitoring PZT combination. One PZT patch acted as an actuator to generate a stress waves, and the other PZT patch was used as a sensor to detect and receive waves propagated through the selected contact surfaces. The propagated energy of the received signal depends on the effective contact area of the contact surfaces, which is ultimately controlled by the tension of the steel strand supplied by a digital jack.

The loading state of the prestressed steel strand can be monitored by analyzing the energy of the received signal based on the wavelet packet method. Due to the property of the PZT patch and the characteristics of the wedge anchorage, once the signal transmission direction of the same PZT combination was interchanged, the energy transmission path was altered, and the received energy was also different. Especially for structures with multiple contact surfaces, when the direct contact surface is selected for prestress monitoring, the signal transmission direction has a significant influence on the propagated energy value. In the test, two signal propagation directions of each PZT combination were compared to determine the optimal transmission mode according to the corresponding contact surface. Monitoring data of two direct contact surfaces shows that the transmitted energy based on wavelet packet method can monitor the tension state of the prestressed steel strand with a wedge anchorage system.

## 2. Contact Model of Wedge Anchorage Based on Hertz Contact Theory

Since the energy monitoring technique based on piezoceramics mainly depends on the energy transferred by the monitored interfaces, it is very important to analyze the change in the effective area of the monitored contact surface. In order to verify the feasibility of the proposed prestress monitoring technique, a 3D finite element model of wedge anchorage was established according to Hertz contact theory to calculate the trend in the contact area between two direct contact surfaces under different tension conditions.

### 2.1. Hertz Contact Theory

**Figure 1:**
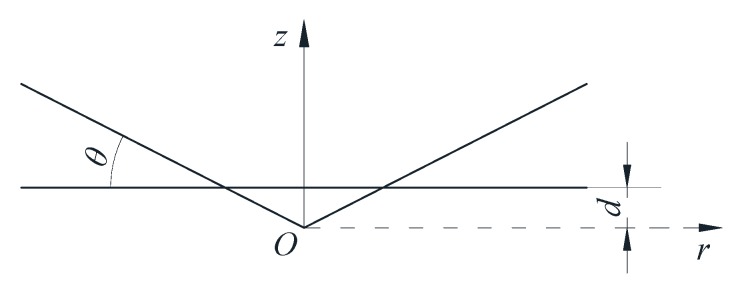
Contact form between rigid conical indenter and elastic half-space body.

Base on Hertz contact theory in contact mechanics, when a rigid cone penetrates an elastic half space body (as shown in Figure 1), the normal force is proportional to the square of the penetration depth:

(1) $$F_{N}=\frac{\pi }{2}E^{*}\frac{d^{2}}{\tan\theta }$$

in which, *F_N_* is the normal force which was perpendicular to the contact surface when the rigid cone and the elastic plane were pressed into each other, *d* is the penetration depth at which the cone was pressed into the elastic space, *θ* is the angle between the cone and the contact surface when the cone and the elastomer are squeezed, and E* is the equivalent elastic modulus of two contact objects.

Equivalent elastic modulus E* is calculated as follows:

(2) $$\frac{1}{E^{*}}=\frac{1-\upsilon_{1}^{2}}{E_{1}}+\frac{1-\upsilon_{2}^{2}}{E_{2}}$$

here, *E*_1_ and *E*_2_ are the elastic modulus of the two elastomers, and *ʋ*_1_ and *ʋ*_2_ are their Poisson’s ratios.

**Figure 2:**
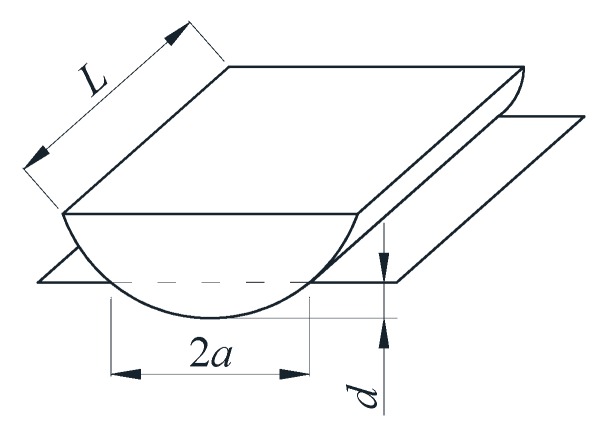
Contact form between rigid cylinder and elastic half space body.

Meanwhile, according to Hertz contact theory when the rigid cylinder is in contact with the elastic half-space body (as shown in Figure 2), the force is linearly proportional to the depth of penetration:

(3) $$F_{R}=\frac{\pi }{4}E^{*}Ld$$

in which, *F_R_* is the normal force which was perpendicular to the contact surface when the rigid cylinder and the elastic plane were pressed into each other, *d* is the penetration depth, *L* is the effective length of the contact area when the cylinder and elastic half space body are squeezed each other, and E* is the equivalent elastic modulus.

### 2.2. Contact Model

**Figure 3:**
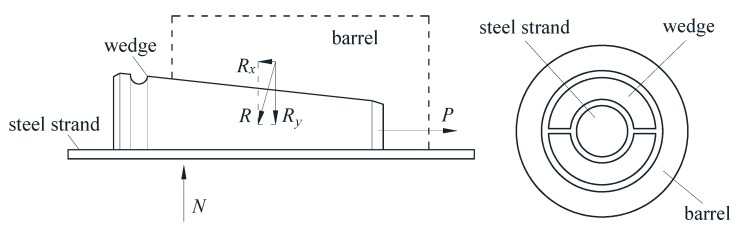
Schematic diagram of stress analysis.

The wedge anchorage system is composed of a barrel, two wedges and a steel strand. Through the interaction between the steel strand and the teeth in wedges, the tension is transmitted to the anchorage, which transmits the force to the tensioned object to bear the load in turn. The force analysis is carried out on the status of the wedges clamping the steel strand and anchoring to the cone hole of the anchorage, as shown in Figure 3.

In Figure 3, *P* is the tensile force of the steel strand, *R* is the acting force of the anchorage on wedges, which can be decomposed into the force *R_x_* along the steel strand direction and the force *R_y_* perpendicular to the steel strand direction. *N* is the interaction force of the steel strand on wedges, so the combined force of the anchorage on wedges is *N* = ∑*R_y_*. Since the steel strand and wedges have a good clamping effect, so the steel strand and wedges can be taken as the same isolation body to analyze their interaction with the anchorage. The overall axial force is balanced, i.e., ∑*R_x_* = *P*.

**Figure 4:**
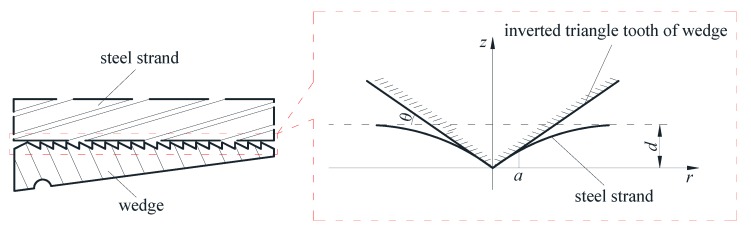
Contact area between steel strand and inverted triangle teeth in wedges.

Since there is an inverted triangle screw tooth on the inside of wedges, it can bite the steel strand during the anchoring processing, as shown in Figure 4.

Based on the above analysis, when the tooth of the inverted triangle bites the steel strand, it is equivalent to the calculation model of the rigid conical indenter pressed into an elastic half-space body. Therefore, the effective contact area between wedges and the steel strand under different tension state can be calculated based on the above assumptions.

According to Hertzian contact theory, when a rigid cone enters an elastic half space body, the relationship between the penetration depth and the contact radius is shown as follows:

(4) $$d=\frac{\pi }{2}a\tan\theta$$

contact area *A* is calculated as follows:

(5) $$A=\pi a^{2}=\frac{4d^{2}}{\pi \tan^{2}\theta }$$

according to Equation (1), then the relationship between *F_N_* and *A* can be expressed as:

(6) $$F_{N}=\frac{\pi^{2}E^{*}\tan\theta }{8}A$$

**Figure 5:**
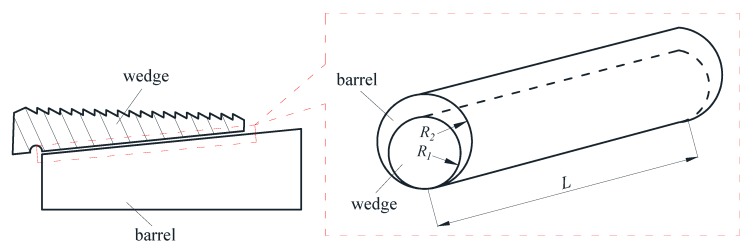
Parallel contact of two cones between wedge and barrel.

In Figure 5, when the tensile force *P* is applied to the steel strand, the force *R* exerted by anchorage on wedges lead to the parallel extrusion between the outer conical surface of wedges and the inner conical surface of the barrel. According to Hertzian contact theory, the squeezing process between wedges and the barrel is equivalent to the parallel contact model of two cylinders.

The contact half width of the two spheres is:

(7) $$a=\sqrt{Rd}$$

equivalent radius *R* of two cylinders:

(8) $$\frac{1}{R}=\frac{1}{R_{1}}+\frac{1}{R_{2}}$$

contact area *A* is calculated as follow:

(9) $$A=2L\sqrt{Rd}$$

based on Equation (3), then the relationship between *F* and *A* can be expressed as:

(10) $$F=\frac{\pi E^{*}}{16RL}A^{2}$$

**Figure 6:**
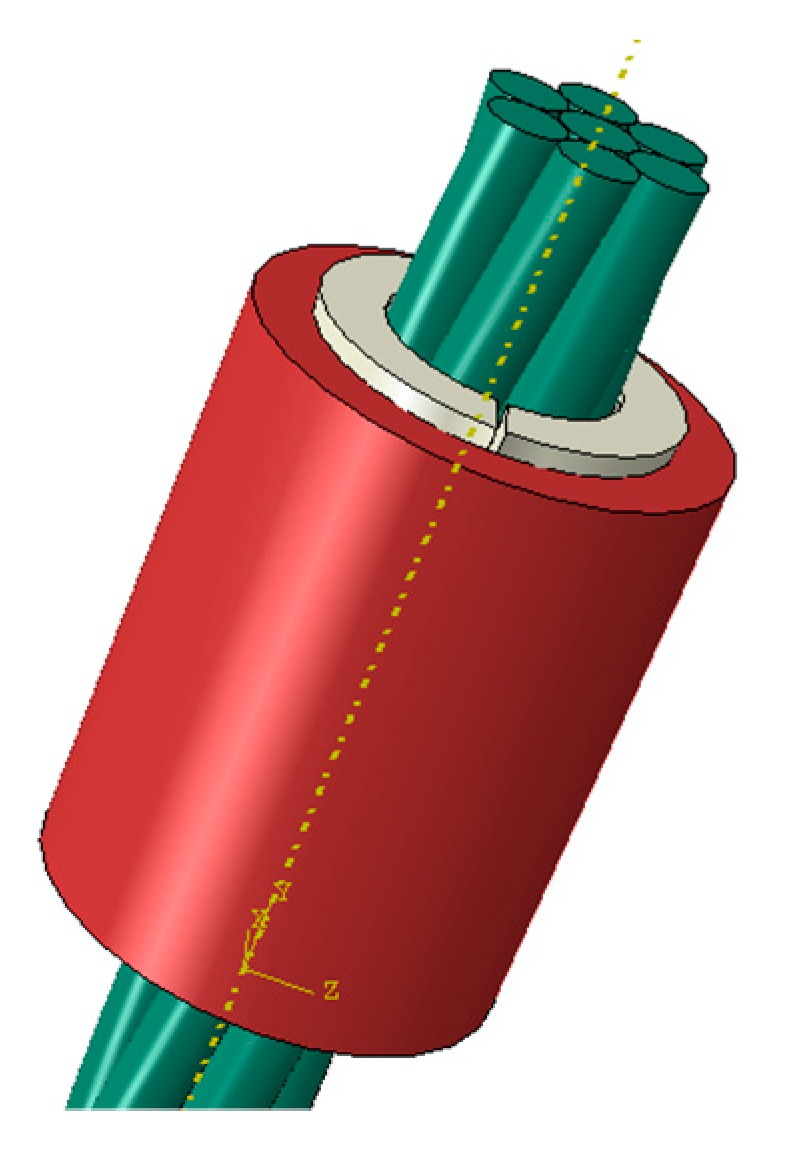
Finite element model of wedge anchorage.

**Figure 7:**
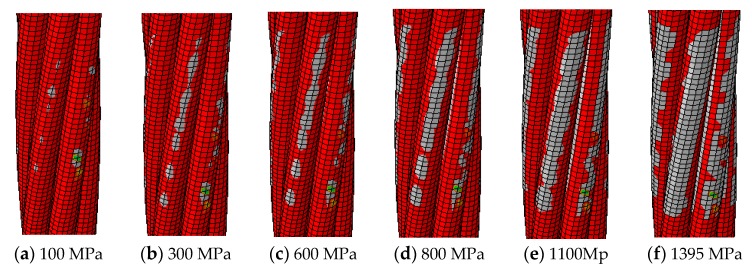
Contact area of the surface of the steel strand (gray surface).

The 3D finite element model of wedge anchorage (as shown in Figure 6) established by Abaqus can further verify the variation of contact surfaces during the tension process of the steel strand. In order to establish the relationship between the monitored contact surface of wedge anchorage and the effective prestress, two different contact areas were calculated under different tension conditions respectively. The steel strand was loaded from 0 MPa to 1500 MPa after the preload was considered, which was divided into 18 working conditions and loaded until the design value. Due to space limitations, only six loading conditions are shown in Figure 7 that can represent the change in contact areas during tensioning.

**Figure 8:**
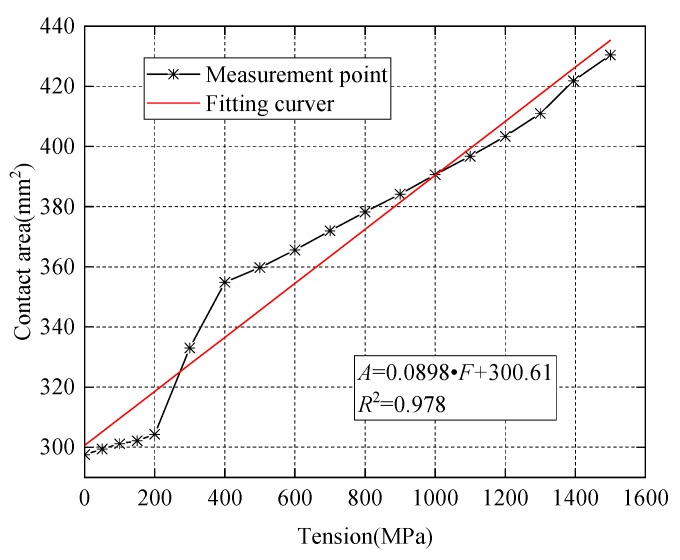
Contact area between steel strand and wedges.

In Figure 7 and Figure 8, the numerical calculation results of the direct contact surface between the steel strand and wedges is consistent with the change of theoretical values. This verified the feasibility of using this contact surface to monitor the anchoring tightness.

**Figure 9:**
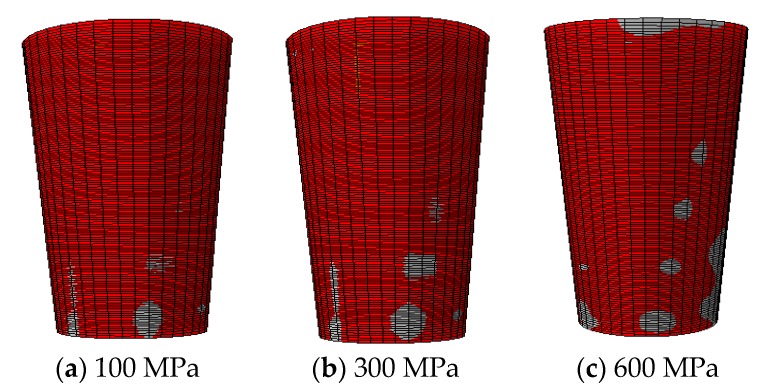

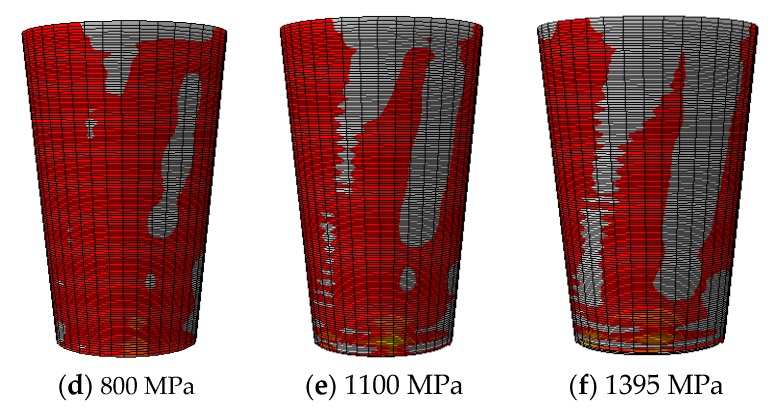
Contact area of the outer side of the wedge (gray surface).

**Figure 10:**
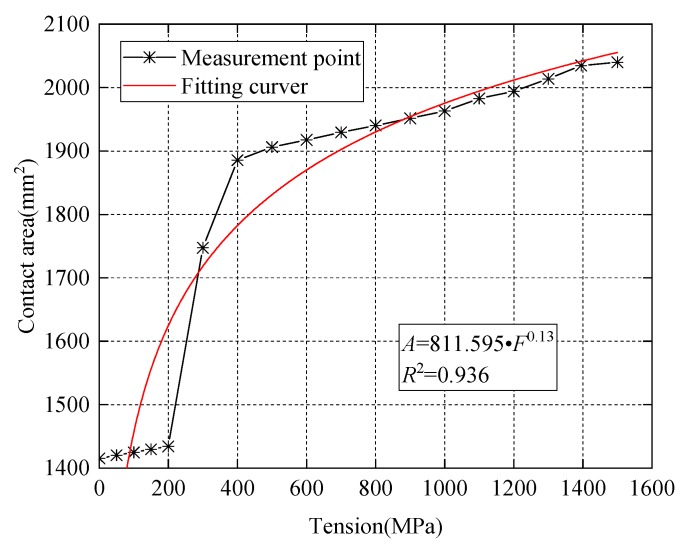
Contact area between barrel and wedges.

In Figure 9 and Figure 10, the parallel contact area of the two cones between wedges and the barrel gradually increases with an increase of the applied loading force. The fitting result of the numerical calculation is consistent with the variation of the theoretical value. It is also verified that this contact surface can be used to monitor the change of the prestress.

## 3. Detection Principle of Anchoring Tightness Based on Wavelet Packet Analysis

### 3.1. Piezoelectric Lead Zirconate Titanate (PZT)-Based Prestress Monitoring with Wedge Anchorage

**Figure 11:**
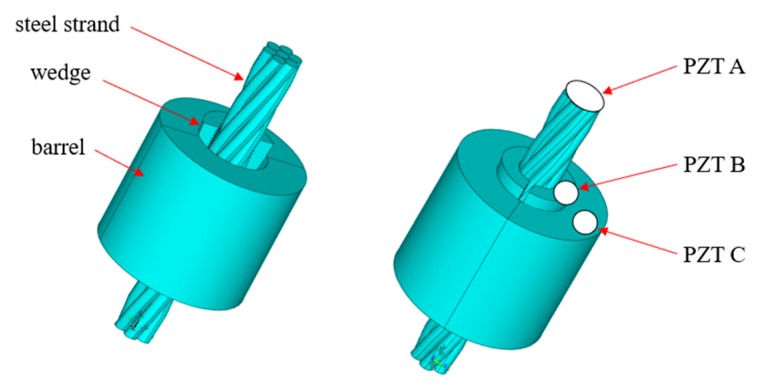
Wedge anchorage system and paste position of lead zirconate titanate (PZT) patches.

The wedge anchorage system consists of a steel strand, two wedges and barrel, as shown in Figure 11. PZT patches were adhered to the corresponding surfaces of the three components by epoxy glue. The PZT patches attached to the steel strand are marked as PZT A; the PZT patches attached to wedges are marked as PZT B; PZT patches pasted on the barrel are labeled as PZT C.

### 3.2. Detection Principle for Wedge Anchorage-Connected Structures Based on Wavelet Packet Analysis

**Figure 12:**
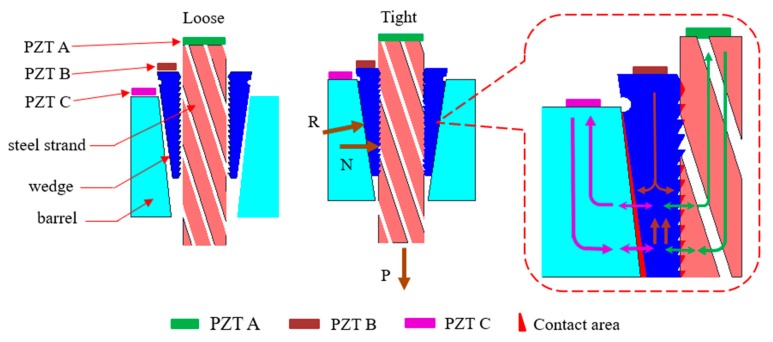
Energy transfer diagram of wedge anchorage.

The wedge anchorage system is the key component for the long-term effective work of prestressed steel strands. When the tensile force P is applied to the steel strand, the wedge would bite the steel strand under the effect of horizontal force N and two rough contact faces between wedge and barrel would be squeezed into each other by the vertical force R, as shown in Figure 12. Due to the three components of wedge anchorage, one contact surface between the steel strand and wedges (AB contact surface) and the other direct contact surface between wedge and barrel (BC contact surface) were formed.

The excited signal from PZT A can be propagated to PZT B through the AB contact surface. Once the signal from PZT A was received by PZT B, it also could flow to PZT C through the BC contact surface to achieve energy transfer across the interface. While the signal from PZT B can be propagated to PZT A through the AB contact surface, it also can flow to PZT C through the BC contact surface to achieve multi-interface transmission of energy. Since PZT combines the characteristics of actuator and sensor, the reverse transmission of energy can be obtained by exchanging the signal transmission direction in the same PZT combination.

The variation of the contact area of two different contact surfaces is consistent with the variation of the loading conditions. With the tension of steel strand increasing, the contact areas of the wedge anchorage system increases, and the energy propagated between two direct contact surfaces is further increased. Determining the relationship between the transmitted energy of two contact surfaces and the loading force, the effective prestress can be monitored.

**Figure 13:**
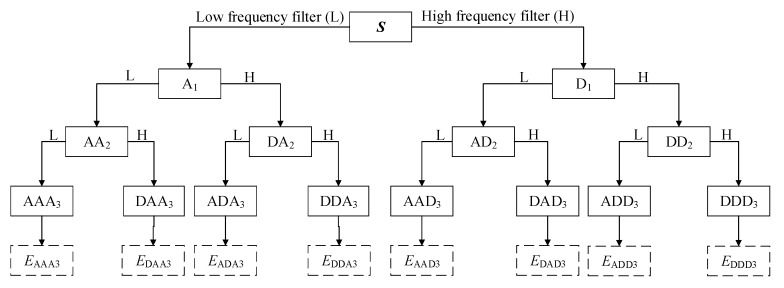
Wavelet packet decomposition process.

Wavelet is a useful tool for non-stationary signal analysis with energy concentrating on time. The general step of decomposition of the time-domain signal ***S*** was illustrated in Figure 13. Wavelet packet decomposition is a stepwise decomposition of the original signal, which divides the signal into low-frequency and high-frequency components. First, signals through the low frequency filter and high frequency filter are decomposed into the approximation (A1) and the detail (D1), respectively. Then, the new approximation and detail coefficient are divided into an approximation and a detail again. In this paper, the transmitted signal is analyzed using wavelet analysis and the transmitted energy is used as an indicator for the anchor connection quality.

The propagated signal ***S*** is a signal set {***S***_1_, ***S***_2_, ⋯, ***S***_2_*^n^*} decomposed by a n-level wavelet packet as described in Equations (11)–(15):

(11) $$S=S_{1}+S_{2}+\cdots +S_{i}+\cdots +S_{2^{n}-1}+S_{2^{n}},\;(i=1,2,\cdots ,2^{n})$$

where ***S****_i_* is the decomposition signal, and *i* is the index (*i* = 1, 2, ⋯, 2*^n^*). In this paper, *n* = 5. ***S****_i_* can be described as follows:

(12) $$S_{i}=\;[s_{i,1}\;\;\;\;\;s_{i,2}\;\;\;\;\cdots \;\;\;\;s_{i,j}\;\;\;\;\cdots \;\;\;\;s_{i,m-1}\;\;\;\;s_{i,m}],\;(j=1,2,\cdots ,m)$$

where *m* is the number of samples. Then, the signal ***S*** can be defined as:

(13) $$E=[E_{1}\;\;\;E_{2}\;\;\;\;\cdots \;\;\;\;E_{i}\;\;\;\;\cdots \;\;\;\;E_{2^{n}-1}\;\;\;\;E_{2^{n}}],\;(i=1,2,\cdots ,2^{n})$$

in which, *E_i_* is the energy of corresponding decomposition signal and is described as:

(14) $$E_{i}=\sum_{j=1}^{m}s_{i,j}^{2},\;\left(i=1,2,\cdots ,2^{n}\right)$$

thus, the energy of signal ***S*** can be expressed as as follows:

(15) $$E=\sum_{i=1}^{2^{n}}E_{i}.$$

Finally, the signal transmitted by two internal direct contact surfaces under different tension conditions of the steel strand, that can be reflected by the wavelet packet energy to achieve the purpose of monitoring the tightness of the steel strand.

## 4. Experimental Setup

**Figure 14:**
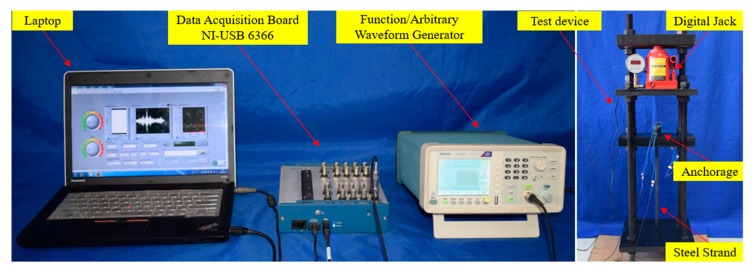
Experimental setup.

**Figure 15:**
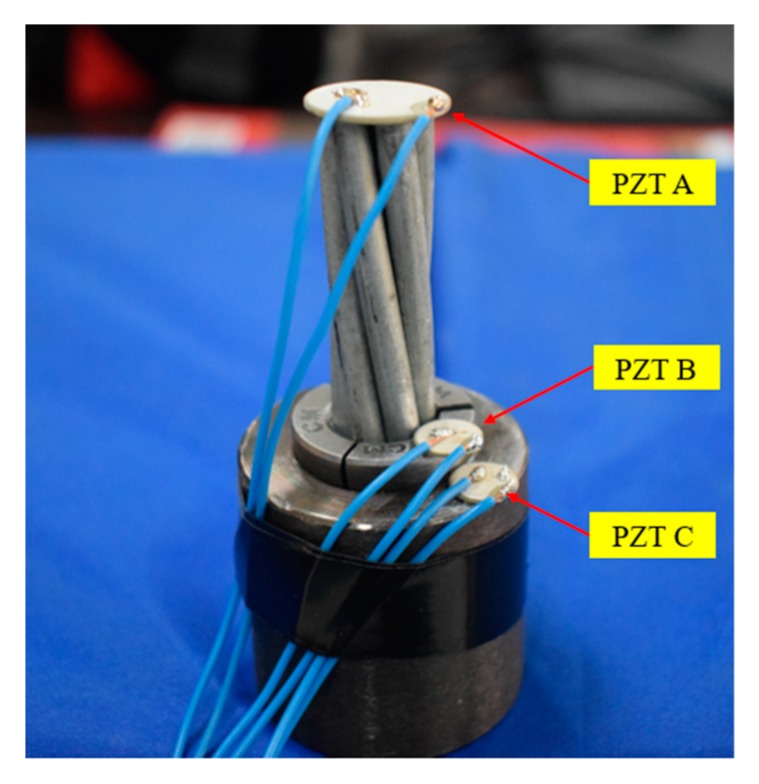
Piezoelectric lead zirconate titanate (PZT) locations of two monitoring techniques.

Experimental equipment includes a National Instrument (NI) data acquisition card, a waveform generator, a computer, and an experimental setup, as shown in Figure 14 and Figure 15. The steel strands were loaded through the digital jack on the reaction frame. Each test was divided into six different loading levels, which were sequentially increased by 1016 N. In order to prevent large relative slip of the wedges during the tensioning process of the steel strand, the initial pretension was usually applied to the steel strand in the actual engineering tensioning process. During this experiment, an initial pretension was applied to the steel strand before loading to ensure that the wedge and the steel strand were embedded in the barrel with the initial contact. According to the structural characteristics of wedge anchorage, the experiment was divided into two different prestress monitoring methods. The first monitoring method was based on the contact surface (AB contact surface) between the steel strand and wedges. The second monitoring method was based on the contact surface (BC contact surface) between wedge and barrel.

During the experiment, when a certain load was applied to the steel strand, the Gaussian pulse generated from the waveform generator and output to PZT A, which was used as actuator. Then the stress wave was detected and received by PZT B, which was used as a sensor, through the contact surface between the steel strand and wedges. Finally, the propagated signal was acquired by the computer via the NI data acquisition card. According to the energy transmitted by the AB contact surface under different loading conditions, the relationship between the tensile force of the steel strand and the wavelet packet energy can be established. In the same way, the energy transmitted by the BC contact surface under different tension conditions can be obtained. Finally, according to the propagated energy based on wavelet packet analysis method under different tension conditions, the prestress can be monitored. In the test, all the propagated signals were analyzed with wavelet packet analysis technique. Additionally, the signal was decomposed into 32 signal sets by a 5-level wavelet packet decomposition.

Corresponding information of PZT patchs is shown in Table 1.

## 5. Experimental Procedure, Results and Analyses

**Figure 16:**
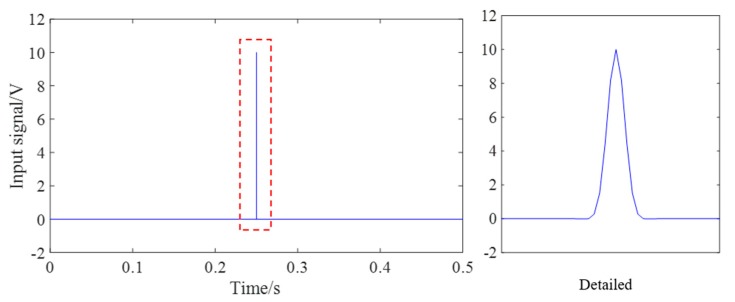
The input signal.

The energy values corresponding to two contact surfaces of the steel strand under six tension conditions were collected, respectively. The frequency sweep from 100 Hz to 500 kHz was applied to each contact surface of wedge anchorage system. One of the received signals and its Fourier spectrum of each contact surfaces were shown below. It was found that the maximum resonance frequency of the energy value appeared in the sweep frequency range, which proved the rationality of the selected sweep frequency range. The input signal was shown in Figure 16.

### 5.1. Prestress Monitoring Based on AB Contact Surface

**Figure 17:**
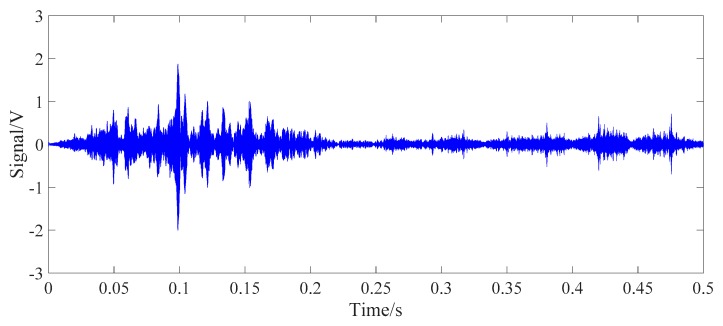

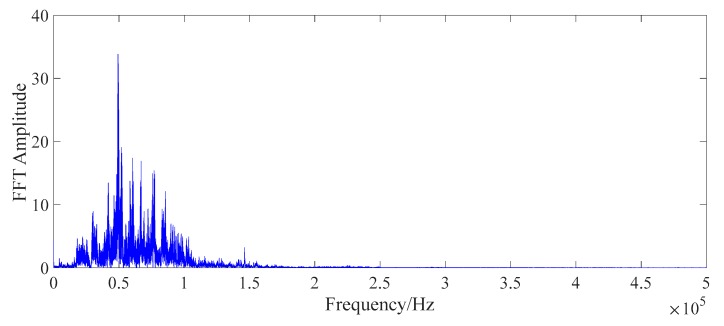
Received signals and its Fourier spectrum of PZT AB transmission mode.

The first prestress monitoring method was through the contact surface (AB contact surface) between the steel strand and wedges. The wavelet packet energies of PZT AB and PZT BA transmission modes were compared under the same loading conditions. In the test, PZT AB combinations under six loading contiditions were performed with a frequency sweep from 100 Hz to 500 kHz. The sweep frequency results shown that there was an obvious resonance frequency in the range of the sweep frequency region, which proved the rationality of the experimental selection frequency band. Due to space limitations, only the sweep result of PZT AB transmission mode is listed in Figure 17.

**Figure 18:**
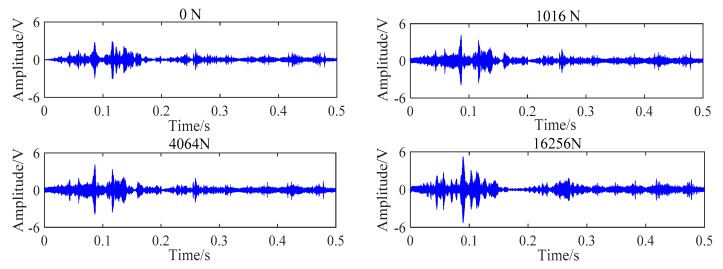
Received signal of PZT BA combination under four loading conditions.

During the experiment, each steel strand was loaded by digital jack, and the loading process was divided into six tension conditions with an interval of 1016 N. With the increase of the load of the steel strand, the amplitude of the corresponding received signal was gradually increased. Due to space limitations, the received signal of PZT BA transmission mode under four tension conditions was randomly selected, as shown in Figure 18.

In order to test the reliability and repeatability of the experiment, five independent experiments were implemented on PZT AB combinations. In each test, the load of the steel strand was started from 0 N with six working conditions. Due to the characteristics of the wedge anchorage system, once the transmission direction of same PZT AB combination was interchanged, that is, the PZT AB and the PZT BA transmission modes have different energy transmission paths, the obtained wavelet packet energy values were also different. At the same time, signal transmission directions of PZT AB and PZT BA combinations were compared with the wavelet packet energy values. The results of five experiments of PZT AB combinations were presented in Table 2. Using the wavelet energy at 0 MPa as the reference value, the relative change values of the transmitted energy under each loading condition were calculated. Then, the difference between the energy value transferred from the maximum loading condition and the reference value was regarded as the absolute change value of the experiment. Finally, the relative change rate was calculated according to the relative change value and absolute change value of the energy received under each loading condition.

**Figure 19:**
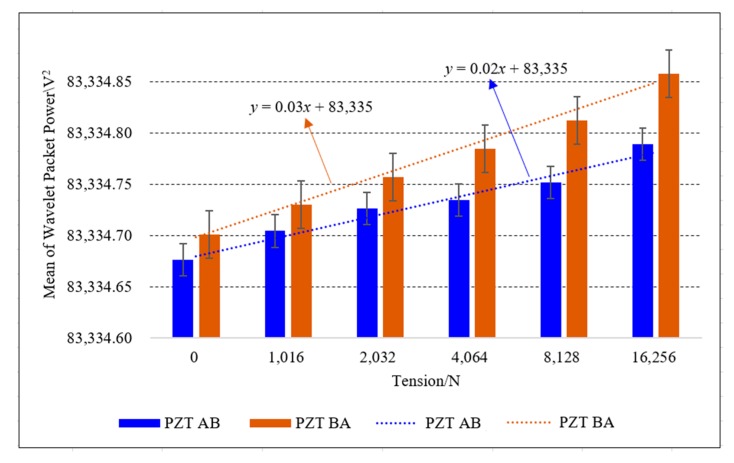
Mean of the wavelet packet energy of two PZT AB combinations.

In Table 2, the transmitted energy of two transmission modes between PZT AB and PZT BA combinations increases with the increase of the load. Experimental data were shown that the loading conditions can be monitored based on the wavelet packet energy of PZT AB combinations. Meanwhile, under the same tension condition of the steel strand, the focused value of PZT BA transmission mode was larger than that of PZT AB transmission mode. In addition, the mean of transmitted energy from two signal transmission directions was compared under the same PZT combination. The minimum value and maximum value in the experiment are included as error bars in Figure 19.

In Figure 19, the mean of wavelet packet energy of five experiments for two PZT AB transmission modes increases as the loading value increases. The wavelet packet energy of two transmission modes of same PZT combination is different, and the difference of transmitted energy increases with the increase of the loading values. The trend lines of the average energy of two PZT AB combinations indicate the feasibility of using this contact surface to monitor the tension state of the steel strand. When PZT AB transmission mode was selected to monitor the loading conditions, the signal generated from PZT B, a part of energy was transmitted to PZT A through the AB contact surface, a part of the energy was transmitted to PZT C through the BC contact surface. While PZT BA transmission mode was chosen to monitor the anchoring tightness of the steel strand, the signal generated from PZT A was propagated directly to PZT B through the AB contact surface. Therefore, when using the AB contact surface to monitor the tension of the steel strand, it is appropriate to select the PZT BA transfer mode.

### 5.2. Prestress Monitoring Based on Wedge–Barrel (BC) Contact Surface

**Figure 20:**
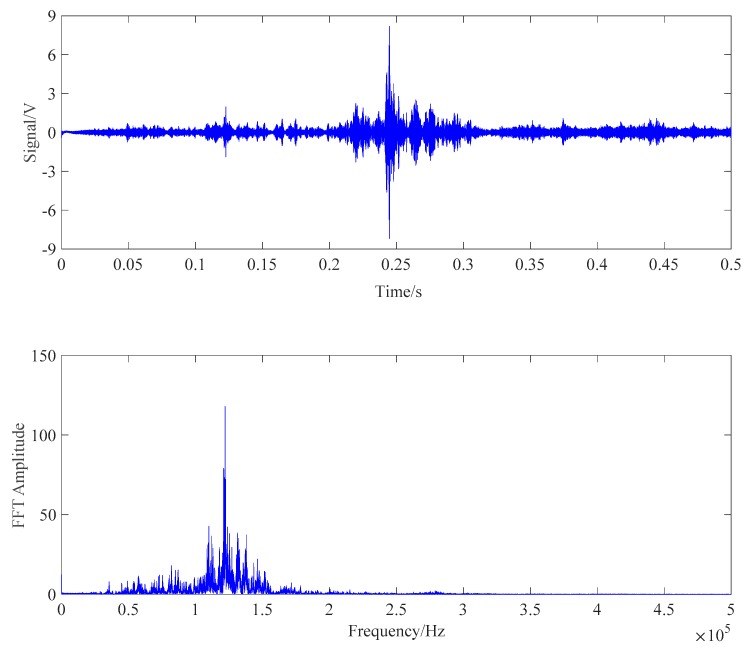
Received signals and its Fourier spectrum of PZT BC combination.

The monitoring method for the tension of the steel strand based on BC contact surface was the same as the AB contact surface monitoring method. First, the reasonable sweep interval was determined by the frequency sweep. Then, wavelet values of PZT BC combinations were compared under different tension conditions. The frequency sweep result of PZT BC transmission mode is shown in Figure 20.

**Figure 21:**
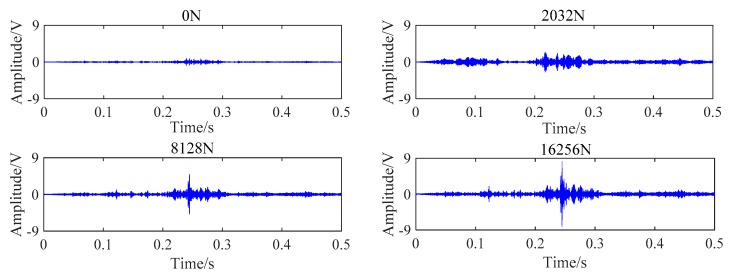
Received signal of PZT BC combination at six loading conditions.

The tension process was also divided into six loading conditions. The test results also shown that the amplitude of the signal transmitted by the BC contact surface increases with the increase of the tension. The change of received signal of PZT BC transmission mode under four loading conditions is shown in Figure 21.

Similarly, the monitoring method based on the BC contact surface was repeated five times. Based on the results of five tests, the average value of the wavelet energy and the relative change ratio of the transmitted energy of the two transmission methods under different loading conditions was compared, as shown in Table 3.

**Figure 22:**
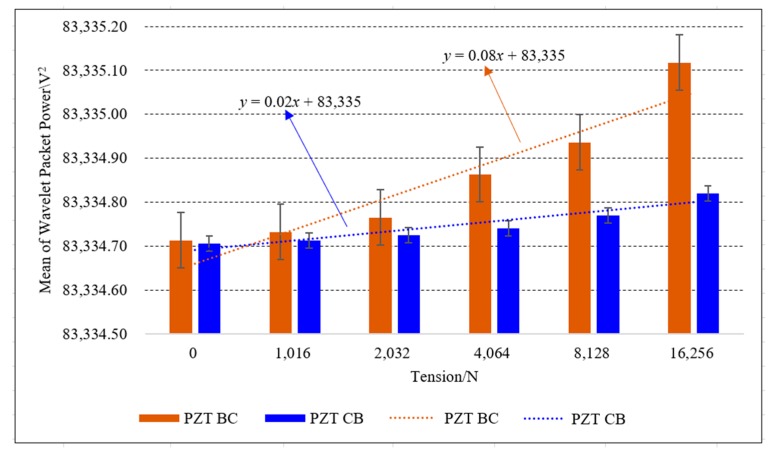
Mean of the wavelet packet energy of two PZT BC combinations.

It can be seen from Table 3 that with the increase of the tension of the steel strand, the mean value of energy propagated by BC contact surface also increases. Moreover, the energy value of PZT BC transmission mode was higher than that of PZT CB transmission mode in the same tension state of steel strand. When the steel strand was loaded step by step, the relative change rate of wavelet energy changed obviously. Experimental trend lines and error bars are included in Figure 22.

In Figure 22, the average value based on the wavelet packet analysis method of five experiments of PZT BC and PZT CB combinations increases as the loading value increases. The wavelet packet energy of two transmission modes of the same PZT combination is different, and the difference increases with the increase of the tensile force. The trend lines of the average energy of two PZT BC combinations indicates the feasibility of using this contact surface to monitor the tension state of the steel strand. When PZT CB transmission mode was selected to monitor the anchoring tightness, the signal generated by PZT B was dispersed and transmitted, a part of energy was transmitted to PZT A through the AB contact surface, and a part of the energy flowed to PZT C through the BC contact surface. While PZT BA transmission mode was chosen to monitor the anchoring tightness, the signal generated from PZT A was propagated directly to PZT B through the AB contact surface. Therefore, when using the BC contact surface to monitor the anchoring tightness of the steel strand, it is recommended to favour the PZT BC transmission mode.

## 6. Conclusions

Monitoring data of two direct contact surfaces of the wedge anchorage system indicated that the wavelet packet energy increases with the increase of anchoring tightness of the steel strand. Meanwhile, since the wedge anchorage system contains multiple contact faces, the path of energy transmission at different contact faces is different. For the two direct contact surfaces between the steel strand-wedge and wedge-barrel, once the signal transfer direction of the same PZT combination is interchanged, the propagated energy value is different. When monitoring the tightness of the steel strand according to the contact surface between steel strand and wedges, PZT BA transmission mode is preferred for better monitoring. When monitoring the tightness of the steel strand according to the contact surface between wedges and barrel, it is more appropriate to choose the PZT BC transmission mode. The main purpose of this paper is to verify the feasibility of the proposed method, and deeper research will be undertaken in the future. The PZT-based wavelet packet analysis method proposed in this paper provides an opportunity for the monitoring of the tensioning of wedge connections with potential future use for the structural health monitoring of wedge connections during loosening.

## Figures and Tables

**Table 1:** Parameters of PZTs.

PZT	Shape	Diameter (mm)	Thickness (mm)	Paste Position
A	round	20	1	steel strand
B	round	10	1	wedge
C	round	10	1	barrel

**Table 2:** Tests results of PZT AB combinations.

Tension/N	PZT AB Combination		PZT BA Combination	
	*μ*/V^2^	Relative Change/ Absolute Change (%)	*μ*/V^2^	Relative Change/ Absolute Change (%)
1016	83,334.7045	24.83	83,334.7299	18.46
2032	83,334.7265	44.41	83,334.757	35.58
4064	83,334.7345	51.57	83,334.7847	53.38
8128	83,334.7521	67.15	83,334.8121	70.85
16256	83,334.7889	100.00	83,334.8578	100.00

**Table 3:** Tests results of PZT BC combinations.

Tension/N	PZT BC Combination		PZT CB Combination	
	*μ*/V^2^	Relative Change/ Absolute Change (%)	*μ*/V^2^	Relative Change/ Absolute Change (%)
1016	83,334.7321	4.79	83,334.7129	6.61
2032	83,334.7651	12.96	83,334.7243	16.55
4064	83,334.8630	37.14	83,334.7396	29.97
8128	83,334.9362	55.21	83,334.7695	56.05
16256	83,335.1174	100.00	83,334.8198	100.00
